# Obituary

**Published:** 1850-03

**Authors:** 


OBITUARY.

The numerous friends of this Journal will be pained to hear of the death of George W. Weissinger, Esq., associate editor and proprietor of the Louisville Daily Journal, and one of the proprietors of the Western Journal of Medicine and Surgery. He was an earnest and devoted friend of medical science, and contributed largely to the success of the medical school of Louisville. He was one of the Trustees of the Louisville University.

Mr. Weissinger was a gentleman of liberal education and of enlarged understanding. He looked with a keen and observant eye upon all the great movements of the day, and, with a hopeful heart, and a powerful pen, he gave his aid to what he considered real progress. Science and literature have lost in him an ardent and useful friend.

